# *Pogostemon cablin* Extract Promotes Wound Healing through OR2AT4 Activation and Exhibits Anti-Inflammatory Activity

**DOI:** 10.3390/cimb46080540

**Published:** 2024-08-21

**Authors:** Jung Ha Choo, Daehyun Kim, Kyoungin Min, So Young Lee, Nae Gyu Kang

**Affiliations:** Science Research Park, LG Household and Healthcare Ltd., Gangseo-gu, Seoul 07795, Republic of Korea; daehyun@lghnh.com (D.K.); kimin@lghnh.com (K.M.); soyounglee@lghnh.com (S.Y.L.); ngkang@lghnh.com (N.G.K.)

**Keywords:** *Pogostemon cablin*, patchouli, cell proliferation, wound healing, olfactory receptor, OR2AT4, anti-inflammation

## Abstract

Skin healing occurs through an intricate process called wound healing which comprises four phases: coagulation and hemostasis, inflammation, cellular proliferation, and remodeling. Chronic wounds often arise because of prolonged or excessive inflammation, which hinders the healing process and wound closure. Despite the recognized efficacy of *Pogostemon cablin* (patchouli) in wound healing, the precise mechanism of action of *Pogostemon cablin* extract (PCE) on inflammation and wound healing remains poorly understood. In this study, we investigated the effects of PCE on cell proliferation and wound healing, as well as its anti-inflammatory activity, using in vitro experiments. We found that PCE increased cell proliferation and expression of the cell proliferation marker Ki67 and accelerated wound healing in human keratinocytes through the activation of OR2AT4. Furthermore, PCE exhibited anti-inflammatory effects by decreasing the levels of pro-inflammatory cytokines interleukin-6 and -8 in lipopolysaccharide-treated and TNF-α-exposed THP-1 and HaCaT cells, respectively. Overall, these findings suggest that PCE holds therapeutic potential by promoting cell proliferation, facilitating wound healing, and exerting anti-inflammatory effects.

## 1. Introduction

The skin, the outermost layer of the human body, serves as a protective barrier and performs various functions, including protection, water retention, thermoregulation, sensation, and immunity. When the skin is damaged, it heals through a process known as wound healing. This process can be broadly divided into four overlapping phases: coagulation and hemostasis, inflammation, cell proliferation, and remodeling [1]. Inflammation can begin shortly after skin injury. At this stage, immune cells are attracted, cytokines and growth factors are released, and various signaling pathways are activated [2]. However, wound healing can be hindered if inflammation becomes excessive or prolonged and results in chronic wounds that do not heal properly. In chronic wounds, there is an imbalance in the ratio of pro- to anti-inflammatory macrophages. In addition, the ability of macrophages to remove dead neutrophils is reduced [3]. Cell proliferation is a crucial process in wound healing. Keratinocytes, the key cellular constituents of the epidermis, play a pivotal role in epidermal regeneration following injury by engaging in a process called reepithelialization. During this phase, basal epidermal keratinocytes migrate, mature, and differentiate, filling the wounds with new cells [4].

Medical plant extracts have traditionally been used for wound healing. The efficacies of several plant extracts and phyto-derived compounds have been scientifically validated using in vitro and in vivo models [5]. Herbal preparations containing flavonoids, alkaloids, saponins, and phenolic compounds demonstrate antimicrobial, anti-inflammatory, antioxidant, cell proliferation, collagen synthesis stimulation, and angiogenic activities in wound management [6]. Recently, the hydroglycolic extract derived from *Cocos nucifera* flour has been shown to significantly enhance keratinocyte migration through the olfactory receptor family 2 subfamily AT member 4 (OR2AT4), which is found in the nasal cavity and has also been identified in various non-olfactory tissues, including skin cells such as keratinocytes [7,8]. In human keratinocytes, OR2AT4 activation modulates intracellular calcium, inositol phosphate, and cyclic adenosine monophosphate (cAMP) levels, thereby suppressing senescence and promoting cell proliferation [9]. Similarly, Sandalore, a synthetic sandalwood-like odorant, stimulates the olfactory receptor OR2AT4 found in human skin and hair follicles. Sandalore promotes wound healing in human keratinocytes and hair growth in human hair follicles by activating OR2AT4 [10,11].

*Pogostemon cablin* (Blanco) Benth., commonly known as patchouli, is a perennial herb belonging to the Lamiaceae family. It is native to South and Southeast Asia, particularly China, Indonesia, and Malaysia [12]. Patchouli can be extracted using various solvents and extraction methods; however, the predominant approach is hydrodistillation, which specifically employs either steam or water. This process allows for the acquisition of patchouli essential oil (PEO). PEO is typically extracted from dried patchouli leaves. The hydrodistillation of fresh leaves of patchouli results in a reduced yield, because approximately 50% of the PEO is enclosed within cells located deep inside the leaves. To release this interior oil, the leaves must undergo a drying process and potentially a gentle fermentation stage, which enhances the permeability of the cell walls surrounding the oil glands. This facilitates more efficient diffusion of PEO from the leaves during steam distillation [13]. PEO is known for its distinctive aroma and has a long history of use in traditional medicine, perfumery, and aromatherapy. PEO has been traditionally employed to address various ailments, such as colds, fever, vomiting, headaches, and stomachaches. Additionally, PEO has been used in aromatherapy to alleviate depression and stress, and as an aphrodisiac [14]. PEO contains marked levels of sesquiterpenes, primarily patchoulol, which is also referred to as patchouli alcohol. Other sesquiterpenes, such as patchoulene, caryophyllene, pogostol, and pogostone, are also found in the oil. Furthermore, PEO also contains minor components, such as α-, β-bulnesene, norpatchoulene, cyclosychellene, and guaiene [15]. Numerous studies have indicated that various components of PEO exhibit diverse pharmacological effects such as protection against inflammation [16,17], combating microorganisms [18], counteracting aging [19], preventing oxidation [20], and inhibiting biofilm formation [21]. Therefore, although the efficacy of the main components of PEO is well-documented, there exists limited research on *Pogostemon cablin* extract (PCE), particularly on its anti-inflammatory and wound healing properties.

In this study, we obtained PCE by extracting the dried leaves of *P. cablin* that had matured in a moist environment. We investigated its wound healing activity and anti-inflammatory properties, as well as the mechanism underlying its therapeutic effects.

## 2. Materials and Methods

### 2.1. Preparation of PCE

Dried leaves of *Pogostemon cablin* (Blanco) Benth. (Lamiaceae) from Indonesia were purchased from HerbMaul (Chung-ju, Republic of Korea) and identified by Dr. Gil Nam Kim from Oriental Herbal Research Lab., LG Household & Healthcare. A voucher specimen (LG002307) was stored at the Oriental Herbal Research Center, LG H&H. The leaves were exposed to 5% moisture relative to the weight of the raw material every 24 h and aged at 50 °C for 120 h. Following the maturation process, the extract was obtained using caprylic/capric triglyceride (Palm-Oleo (Klang) Sdn. Bhd., Selangor, Malaysia) as a solvent at a ratio of 1:20 with respect to the dry weight of leaves. The extraction was carried out at 25 °C for 3 days.

### 2.2. GC-MS Analysis

GC-MS analyses were performed using a 7890B Agilent 19091S-436 HP-5MS capillary column (60 m × 0.25 mm × 0.25 μm; Agilent, Santa Clara, CA, USA) equipped with a 5977B mass spectrometer (Agilent). Helium was used as a carrier with a constant flow velocity of 1 mL/min. The oven temperature was maintained at 80 °C for 5 min, then ramped up at a rate of 8 °C per minute until it reached 320 °C. It was then held at 320 °C for 20 min. Compound identifications were conducted by comparing mass spectral data from the Agilent library based on the NIST database. This was validated through the comparison of spectral data and retention time of the patchouli alcohol standards (Sigma-Aldrich, St. Louis, MO, USA).

### 2.3. Cell Culture

Human immortalized keratinocytes (HaCaT) were acquired from AddexBio (San Diego, CA, USA), and the human monocytic cell line THP-1 was obtained from American Type Culture Collection (ATCC; Manassas, VA, USA). HaCaT cells were grown in Dulbecco’s modified Eagle’s medium (DMEM; Gibco, Thermo Fisher Scientific, Waltham, MA, USA) and THP-1 cells were cultured in Roswell Park Memorial Institute 1640 medium (RPMI1640; Gibco) supplemented with 0.05 mM 2-mercaptoethanol. Both cell lines were supplemented with 10% fetal bovine serum (FBS; Gibco) and 1% antibiotic–antimycotic solution (Gibco) and were incubated in a humidified environment with 5% carbon dioxide (CO_2_) at a temperature of 37 °C.

### 2.4. Cell Cytotoxicity Assay

Cell cytotoxicity was measured using a Cytotoxicity Lactate Dehydrogenase (LDH) Assay Kit-WST (Dojindo Molecular Technologies, Rockville, MD, USA). HaCaT cells (1 × 10^5^ cells/well) were seeded into 96-well plates and cultured for 24 h. On the following day, the cells were washed with phosphate-buffered saline (PBS; Solbio, Seoul, Republic of Korea) and treated with different concentrations of PCE in serum-free DMEM for 24 h. Lysis buffer was added to wells that were not treated with any substance, which served as a positive control. Subsequently, 30 µL of supernatant from each well, including the positive control, was transferred to another 96-well plate. An equal amount of LDH reagent was added, and the mixture was incubated for 30 min. To stop the reaction, 10 µL of the stop solution from the kit was added and the absorbance of samples was measured at a wavelength of 490 nm using a SYNERGY H1 microplate reader (BioTek, Winooski, VT, USA).

### 2.5. Cell Proliferation Assay

Cell viability was assessed using a cell counting kit-8 assay (CCK-8; Dojindo Molecular Technologies). The cells were seeded at a density of 1 × 10^5^ cells in 96-well plates and incubated for 24 h. Subsequently, the cells were rinsed with PBS and exposed to various concentrations of PCE, Sandalore (Givaudan, Vernier, Switzerland), or Oxyphenylon (Sigma-Aldrich) in serum-free DMEM for 24 h. The culture medium was then supplemented with a CCK-8 solution (10%) and the cells were placed in an incubator at 37 °C for 30 min. The absorbance of the samples was then determined at 450 nm using a SYNERGY H1 microplate reader (BioTek).

### 2.6. RNA Extraction and Quantitative Polymerase Chain Reaction (qPCR)

THP-1 cells (3 × 10^5^ cells/well) and HaCaT (2 × 10^5^ cells/well) were seeded in 12- and 24-well culture plates, respectively, and cultured for 24 h. After incubation, THP-1 cells were treated overnight with phorbol 12-myristate 13-acetate (PMA) (50 ng/mL). Thereafter, the cells were rinsed with PBS and exposed to either vehicle- or PCE-containing serum-free DMEM for 24 h. The cells were then stimulated with *E. coli* lipopolysaccharide (LPS; Sigma-Aldrich) for an additional 24 h. Similarly, HaCaT cells were rinsed with PBS and then treated with serum-free DMEM containing either vehicle or PCE for 1 h, followed by stimulation with TNF-α (Sigma-Aldrich) for 24 h. An RNeasy mini kit (QIAGEN GmbH, Hilden, Germany) was used to extract total RNA from THP-1 and HaCaT cells according to the manufacturer’s protocol. The concentration and purity of RNA were evaluated using a NanoDrop 2000 spectrophotometer (Thermo Fisher Scientific). Subsequently, a cDNA synthesis kit (Philekorea, Seoul, Republic of Korea) was used to reverse-transcribe 1 µg of RNA, and qPCR was conducted using the StepOnePlus^®^ Real-Time PCR System (Applied Biosystems, Waltham, MA, USA) and the following TaqMan probes: proliferation marker Ki-67 (MKI67; Hs04260396_g1), interleukin 6 (Il6; Hs00174131_m1), C-X-C motif chemokine ligand 8 (CXCL8; Hs00174103_m1), and glyceraldehyde-3-phosphate dehydrogenase (GAPDH; #4352665; Thermo Fisher Scientific). mRNA levels were determined using the 2^–∆∆Ct^ method, and relative mRNA levels were determined by normalizing to *GAPDH* levels.

### 2.7. Immunofluorescence Analysis

HaCaT cells were seeded into 24-well plates at a density of 2 × 10^5^ cells/well and incubated overnight. The cells were then washed with PBS and treated with different media: one containing vehicle, another containing PCE, and the other containing PCE and Oxyphenylon for 24 h. After treatment, the cells were fixed using a 4% formaldehyde solution (Biosesang, Seongnam, Republic of Korea), permeabilized with 0.1% Triton X-100, and blocked with a solution containing 5% FBS and 1% BSA for 1 h. Cells were then incubated with monoclonal anti-mouse antibody against Ki67 (1:500; Abcam, Cambridge, UK) and Alexa Fluor™ 568 goat anti-rabbit antibody (1:1000; Thermo Fisher Scientific) in sequence. The cell nuclei were stained with 1 μg/mL 4′,6-diamidino-2-phenylindole (DAPI; Sigma-Aldrich), and immunofluorescence was observed using the EVOS ^TM^ FL Auto2 imaging system (Thermo Fisher Scientific). The number of Ki67-positive cells was determined by analyzing the merged images of Ki67 and DAPI.

### 2.8. Wound Healing Assay

HaCaT cells (2.0 × 10^4^ cells/well) were seeded in a Culture-Insert 2 Well 24 plate (Ibidi GmbH, Gräfelfing, Germany) and cultured overnight. After incubation, the inserts were removed and the cells were treated with PCE, Sandalore, and Oxyphenylon diluted in DMEM containing 5% FBS and 1% antibiotic–antimycotic solution for 18 h. All images were acquired using the EVOS^TM^ FL Auto2 imaging system (Thermo Fisher Scientific). Wound recovery areas were analyzed using Image J bundled with 64-bit Java 8 (https://imagej.net/ij/, accessed on 5 January 2024, NIH, Bethesda, MD, USA).

### 2.9. cAMP-Glo^TM^ Assay

HaCaT cells were seeded in 96-well plates at a density of 1.5 × 10^4^ cells/well and incubated for 24 h. The cells were rinsed with PBS and subsequently treated with vehicle, PCE, or Oxyphenylon for 10 min. To quantify the level of cAMP in the cells, the cAMP-Glo^TM^ Assay (Promega, Madison, WI, USA) was used according to the manufacturer’s instructions. A standard curve was constructed using known concentrations of cAMP. This curve facilitated the correlation of the luminescence signals from the samples with their corresponding cAMP concentrations.

### 2.10. Enzyme-Linked Immunosorbent Assay (ELISA)

THP-1 and HaCaT cells were seeded in 24-well plates (2 × 10^5^ cells per well) and incubated for 24 h. After washing the cells with PBS, they were treated with either vehicle- or PCE-containing serum-free DMEM for 24 h. Following the 24-hour incubation period, the cell culture supernatants were collected and the concentrations of interleukin (IL)-6 and IL-8 were determined using the IL-6 and IL-8 DuoSet ELISA kits (R&D Systems, Minneapolis, MN, USA), respectively, according to the manufacturer’s instructions.

### 2.11. Statistical Analysis

Statistical comparisons between groups were conducted using the Student’s *t*-test in Microsoft Excel 2016. The level of statistical significance was set at *p* < 0.05.

## 3. Results

### 3.1. Patchouli Alcohol Was the Main Component in PCE

**Figure 1:**
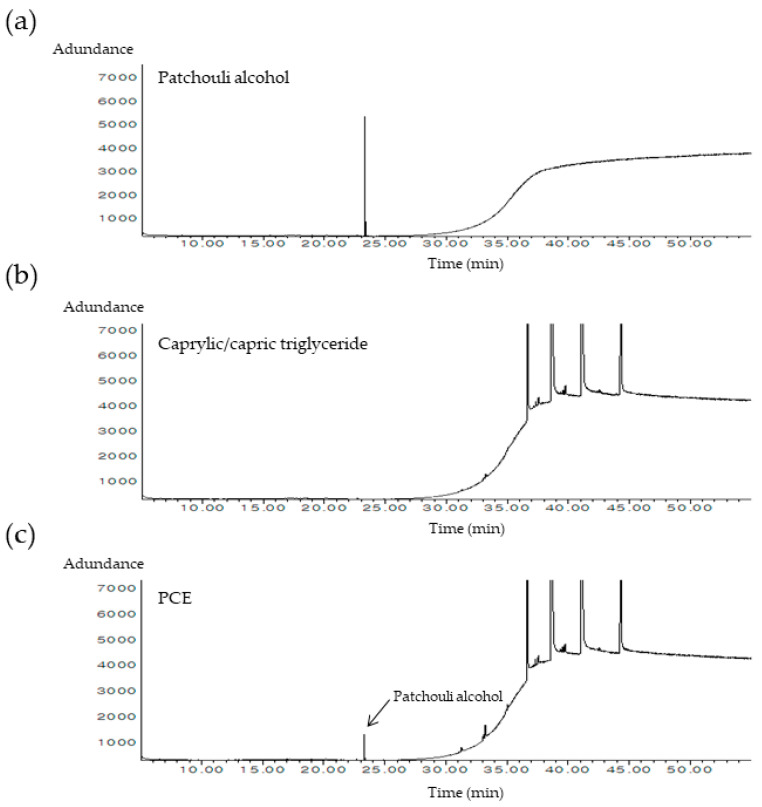
GC-MS chromatograms of PCE. (**a**) Patchouli alcohol standard. (**b**) Extraction solvent; caprylic/capric triglyceride. (**c**) PCE.

To identify the major components of PCE, we conducted a GC-MS analysis. Patchouli alcohol was identified as the primary component in PCE through a library search, and its authenticity was verified by comparing it with a patchouli alcohol standard reference (Figure 1a,c). The peaks observed after 30 min in the analysis were attributed to the solvent, as confirmed by analyzing only caprylic/capric triglyceride (Figure 1b,c). Therefore, patchouli alcohol was the primary component in PCE, similar to PEO.

### 3.2. PCE Increased the Proliferation of HaCaT Cells

**Figure 2:**
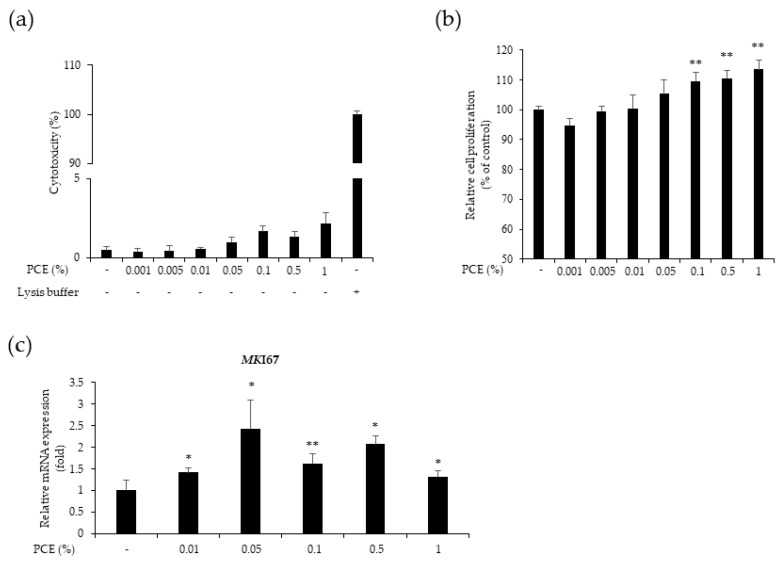
Effect of PCE on the cytotoxicity and proliferation of HaCaT cells. The cells were treated with PCE at different concentrations (0.001, 0.005, 0.01, 0.1, 0.5 and 1%) for 24 h. Cell cytotoxicity (**a**) and cell proliferation (**b**) assessed using the LDH (lactate dehydrogenase) assay and the cell counting kit (CCK)-8 assay, respectively. (**c**) The expression level of cell proliferation marker *MKI67* gene increased by PCE treatment. Quantitative polymerase chain reaction (qPCR) was used to measure the relative expression levels of the *MKI67* gene. Data represent the mean ± SEM. * *p* < 0.05, and ** *p* < 0.01 compared to the control group.

To investigate whether PCE affected the viability of HaCaT cells, we administered various concentrations of PCE (0.001, 0.005, 0.01, 0.05, 0.1, 0.5, and 1%) to the cells and conducted LDH and CCK-8 assays. Even at the highest concentration of PCE (1%), no cytotoxicity was observed in the cells (Figure 2). Additionally, the CCK-8 assay results confirmed that PCE strongly enhanced cell proliferation in a concentration-dependent manner. There was a discernible increase in cell proliferation, starting at a PCE concentration of 0.01%. Significant increases were observed at PCE concentrations of 0.1, 0.5, and 1% (*p* < 0.01). Based on these findings, we chose a PCE concentration of 0.01% for subsequent experiments.

In addition, we performed qPCR to examine changes in the expression of the cell proliferation marker *MKI67*. The expression of the *MKI67* gene in HaCaT cells significantly increased at different concentrations (*p* < 0.05, *p* < 0.01). Specifically, treatment with 0.05% PCE resulted in a significant 2.4-fold increase in *MKI67* mRNA expression compared to the control (*p* < 0.05) (Figure 2c).

### 3.3. Oxyphenylon Attenuated the Cell Proliferation Enhanced by PCE

**Figure 3:**
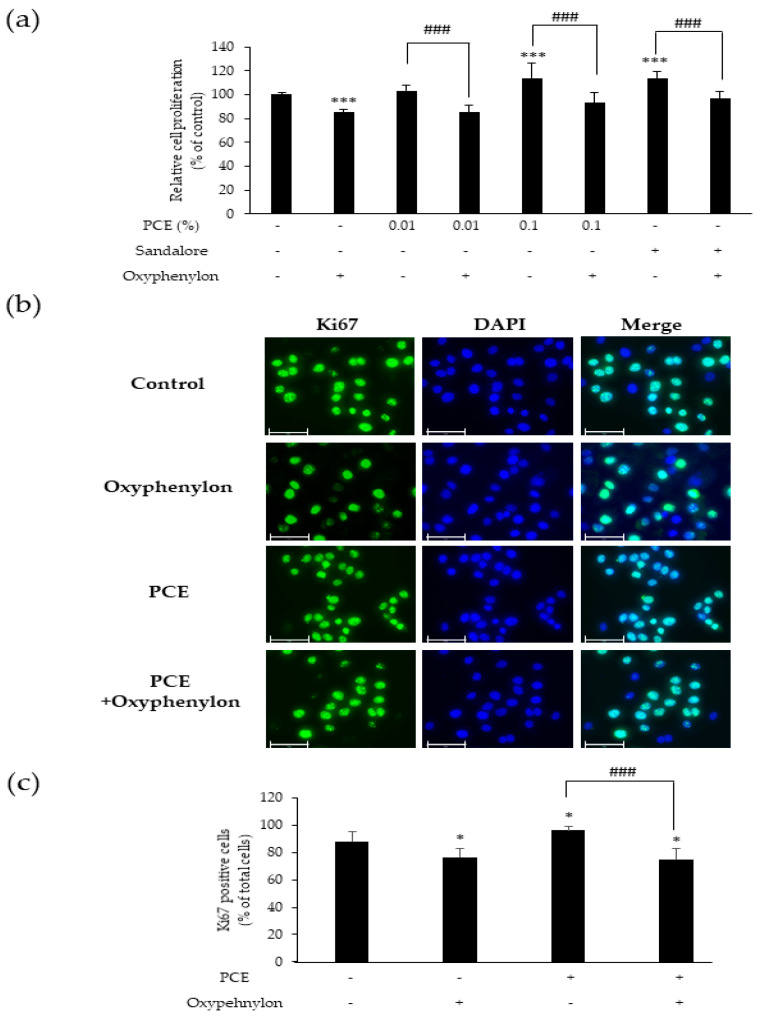
Oxyphenylon attenuated PCE-induced cell proliferation enhancement in HaCaT cells. The cells were treated with PCE (0.01 and 0.1%), Sandalore (500 µM), and Oxyphenylon (500 µM) as indicated for 24 h. (**a**) Cell proliferation evaluated using CCK-8 assay. (**b**) Fluorescent images of HaCaT cells treated with PCE (0.1%) and Oxyphenylon (500 µM) for 24 h, fixed in 4% formaldehyde, permeabilized with 0.1% Triton X-100, and stained with an antibody against Ki67 (green). Nuclei were stained with DAPI (blue). The scale bar size is 75 µm. (**c**) Quantification of Ki67-positive cells by calculating the proportion of Ki67-positive cells out of the total cell count, based on the stained merged image. Data represent the mean ± SEM. * *p* < 0.05 and *** *p* < 0.001 compared to the control, ### *p* < 0.001 compared to the Oxyphenylon-treated group.

Olfactory receptors, referred to as smell receptors, are responsible for detecting and recognizing different odors or scent molecules present in our environment. The ectopic expression of olfactory receptors in numerous non-sensory tissues is well-known [8]. *Cocos nucifera* extract and Sandalore, a synthetic sandalwood aroma chemical, activate the olfactory receptor OR2AT4 and promote cell proliferation and wound healing in keratinocytes [7,10]. *P. cablin* is an aromatic medicinal herb and PCE contains various fragrant components. Furthermore, based on our observation of increased cell proliferation by PCE (Figure 2b), we speculated that PCE may affect OR2AT4. To confirm this hypothesis, we first performed a CCK-8 assay using PCE, Sandalore as an OR2AT4 agonist, and Oxyphenylon as an OR2AT4 antagonist. We found that treatment with PCE (0.01 and 0.1%) in HaCaT cells led to a significant dose-dependent increase in cell proliferation compared to the control group (*p* < 0.001), as also previously observed (Figure 2b). Moreover, treatment with 500 µM Sandalore, which served as a positive control, showed a significant increase in cell proliferation. However, co-treatment with PCE and 500 µM Oxyphenylon resulted in a considerable decrease in cell growth compared to with PCE alone (*p* < 0.001) (Figure 3a).

We also performed immunofluorescence analysis to determine whether Oxyphenylon suppressed the elevated expression of the proliferation marker Ki67 induced by PCE. Immunofluorescence analysis revealed a 7.5% increase in the number of Ki67-positive cells in the 0.1% PCE-treated group compared to the control group. In contrast, cotreatment with 0.1% PCE and 500 µM Oxyphenylon resulted in a significant 20% drop in the proportion of Ki67-positive cells compared to treatment with PCE alone (*p* < 0.001) (Figure 3b,c). These results suggest that PCE stimulates the proliferation of HaCaT cells, possibly through the activation of OR2AT4.

### 3.4. PCE Accelerates Wound Healing through OR2AT4 Activation

**Figure 4:**
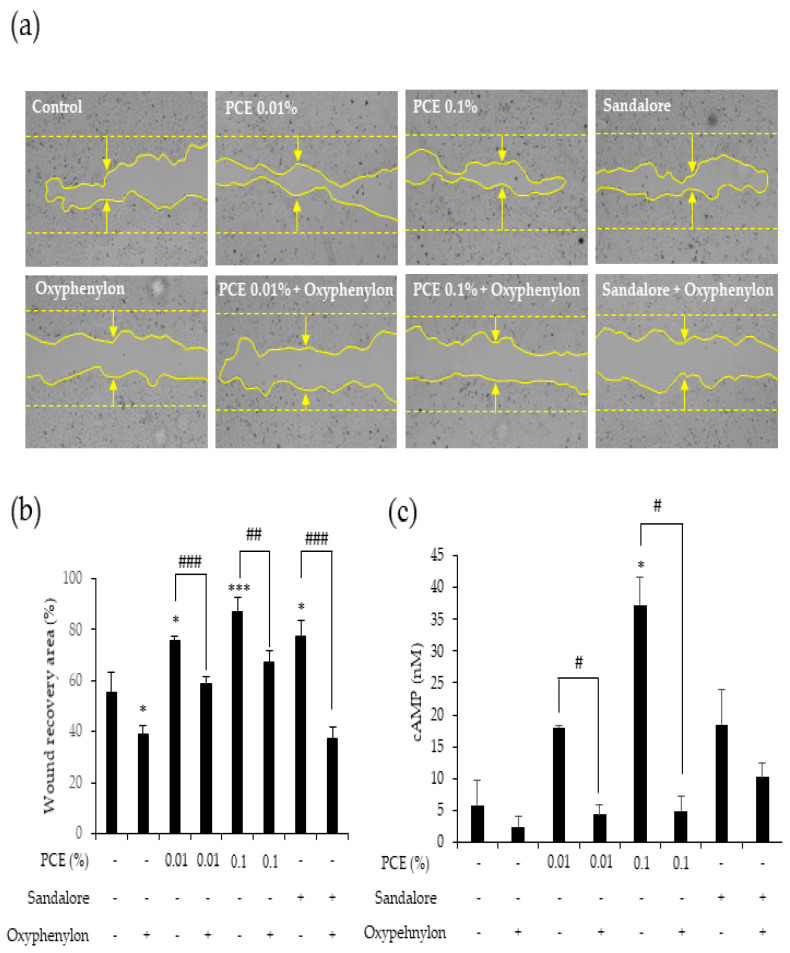
PCE induced wound healing through the activation of OR2AT4 in vitro. (**a**) An artificial wound was created using a Culture-Insert 2 Well 24 plate (ibidi) for the wound healing assay. All wound areas were initially equal in size before treatment, and the wound area was measured after 18 h of treatment with PCE (0.01 and 0.1%), 500 µM Sandalore, and 500 µM Oxyphenylon. (**b**) Graphical representation of the area of wound healing in the image (**a**). (**c**) Graph showing PCE-induced dose-dependent increase in cAMP levels in HaCaT cells treated with PCE, 500 µM Sandalore, and 500 µM Oxyphenylon for 20 min. Data represent the mean ± SEM. * *p* < 0.05 and *** *p* < 0.001 compared to the control; # *p* < 0.05, ## *p* < 0.01, and ### *p* < 0.001 compared to the Oxyphenylon-treated group.

We examined the effects of PCE on wound healing in HaCaT cells. The scratch wound healing assay demonstrated that PCE reduced the wound area in a concentration-dependent manner. Specifically, the group treated with 0.1% PCE exhibited a significantly improved wound healing rate (79%) (*p* < 0.01). Similarly, the group treated with Sandalore at a concentration of 500 µM demonstrated a marked improvement in wound healing (77%) (*p* < 0.05). In contrast, co-treatment of PCE and Oxyphenylon hampered the wound healing process (*p* < 0.1 and *p* < 0.001), which was comparable to the outcome observed with the combined administration of Sandalore and Oxyphenylon (*p* < 0.01) (Figure 4a,b). These findings suggest that the effects of PCE on cell proliferation and wound healing are mediated through OR2AT4.

To confirm whether PCE activates OR2AT4, we assessed cAMP release in HaCaT cells after PCE treatment at concentrations of 0.01% and 0.1%, as well as 500 µM Oxyphenylon treatment alone or in combination with PCE. PCE induced a concentration-dependent increase in cAMP secretion. Additionally, Oxyphenylon significantly inhibited (*p* < 0.05) this PCE-induced increase in cAMP. This indicates that the PCE-induced cAMP secretion was dependent on the activation of OR2AT4 (Figure 4c). Overall, PCE acted on OR2AT4, leading to increased cAMP secretion, enhanced cell proliferation, and accelerated wound healing in HaCaT cells.

### 3.5. PCE Exhibited Anti-Inflammatory Activity in THP-1 and HaCaT Cells

**Figure 5:**
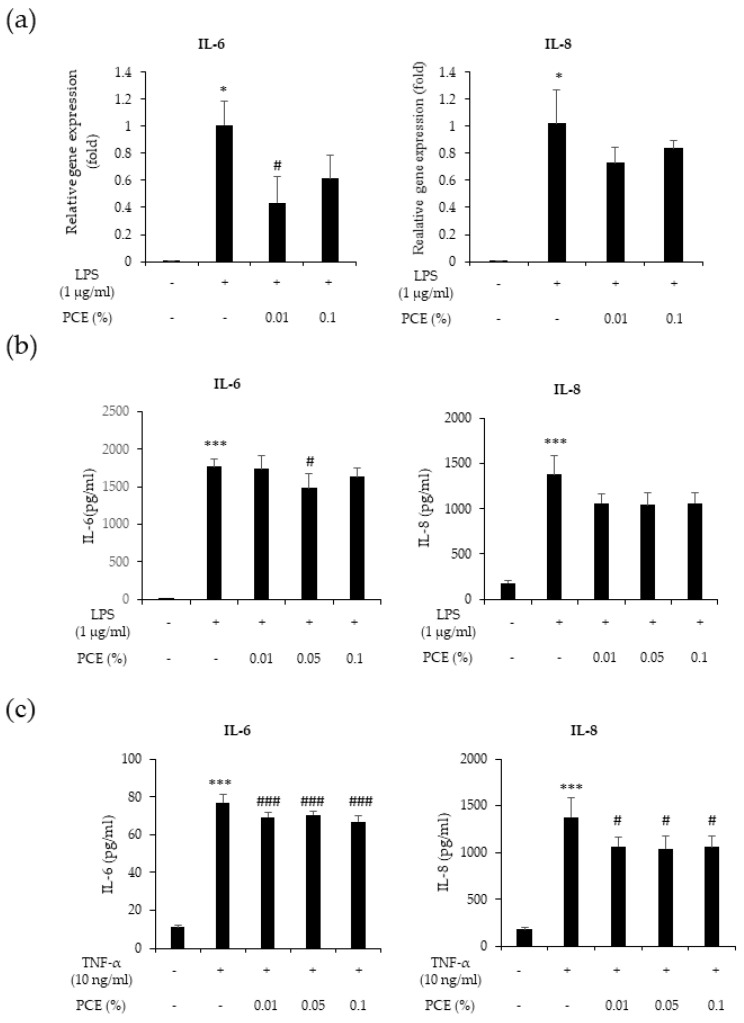
Anti-inflammatory activities of PCE in THP-1 and HaCaT cell. THP-1 and HaCaT cells were pretreated with various concentrations of PCE (0.01, 0.05, and 0.1%) and then stimulated with LPS and TNF-α for 24 h. (**a**) mRNA expression levels of inflammatory genes IL-6 and IL-8 measured using qPCR. (**b**,**c**) Protein levels of pro-inflammatory cytokines IL-6 and IL-8 in THP-1 (**b**) and HaCaT (**c**) cells measured using ELISA. The protein levels of each protein were normalized to the total protein amount. Data represent the mean ± SEM of three independent experiments. Statistical significance is indicated as * *p* < 0.05 and *** *p* < 0.001 compared to the control group; # *p* < 0.05 and ### *p* < 0.001 compared to the LPS and TNF-α treated group.

The generation of inflammatory cytokines, and their subsequent elimination by macrophages, is linked to the wound healing process. To evaluate the potential anti-inflammatory effects of PCE, we first performed qPCR for pro-inflammatory cytokines IL-6 and IL-8. As shown in Figure 4a, PCE reduced the LPS-stimulated increased mRNA levels of *IL-6* and *IL-8* genes in THP-1 cells. We also performed ELISA on LPS-stimulated THP-1 and HaCaT cells exposed to TNF-α. Administration of PCE resulted in a significant decrease in the production of the pro-inflammatory cytokines IL-6 and IL-8 in TNF-α-activated THP-1 and HaCaT cells (*p* < 0.05, *p* < 0.001) (Figure 5b,c). These results suggest that PCE exhibits anti-inflammatory properties by suppressing the production of inflammatory cytokines, such as IL-6 and IL-8.

## 4. Discussion

Throughout history, patchouli has been widely used for its distinctive and enthralling scent. Patchouli-derived essential oils and extracts possess a unique and recognizable fragrance characterized by an aromatic, subtle, herbal, woody, and minty aroma. Consequently, it is frequently employed as a popular scent in the perfume and cosmetics industries [13]. In this study, we utilized caprylic/capric triglycerides for patchouli extraction to obtain a PCE. We identified that the major component in PCE was patchouli alcohol through GC-MS analysis. We also examined the proliferative effects of PCE on keratinocytes and wound healing and the potential mechanism underlying its effects. We found that the effects of PCE are mediated by the activation of OR2AT4. In addition, we observed the anti-inflammatory effects of PCE.

*P. cablin* is predominantly extracted as an essential oil, and numerous studies have focused on its composition. Previous studies investigating the constituents of PEO primarily focused on volatile and low-molecular-weight compounds, particularly patchouli alcohol, which was widely recognized as the predominant component [14]. However, studies focusing on the chemical composition of PCE are limited. One study compared the composition of PCE obtained using two different traditional methods: maceration with 95% ethanol and decoction with water. The results revealed a higher total polyphenol content in the water extract and a higher total flavonoid content in the ethanol extract [22]. Compared to PEO, PCE may contain a wider range of phytochemicals, including non-volatile compounds such as flavonoids, alkaloids, polyphenols, and glycosides. Another study analyzed the non-volatile constituents of a 60% methanol extract of *P. cablin* and identified flavonoids as the predominant components alongside organic acids, phenylpropanoids, sesquiterpenes, and alkaloids. Notably, patchouli alcohol, which is a sesquiterpene and primary constituent of PEO, was absent in this methanol extract of *P. cablin* [23]. Therefore, the composition of PCE may vary depending on the extraction method. In this study, as in the PEO analysis, patchouli alcohol was identified as the main component in PCE. This is attributed to the use of a relatively non-polar solvent, caprylic/capric triglyceride.

Wound healing involves the proliferation of cells, including keratinocytes. Keratinocytes are skin cells that play a crucial role in re-epithelialization and the formation of a functional epidermis during wound healing. The proliferation and migration of keratinocytes are necessary for rapid wound closure and tissue homeostasis restoration [4]. CCK-8 and scratch assays can be used to assess cell proliferation and migration in vitro during wound healing [24,25]. Our findings demonstrate that PCE enhances keratinocyte proliferation and facilitates wound healing. We also observed that PCE increases the mRNA expression of the *MKI67* gene, a marker of cell proliferation, confirming its ability to promote keratinocyte proliferation. Previous studies have shown that patchouli alcohol, a major component of PEO, accelerates wound healing in obese mice [26]. Additionally, PEO has been incorporated into biocompatible coatings for wound dressings, demonstrating antimicrobial properties and the ability to prevent biofilm development [27]. Therefore, our results reveal that PCE has therapeutic potential for wound healing by promoting cell proliferation and migration.

To further elucidate the mechanisms underlying PCE-induced cell proliferation and wound healing, we focused on the olfactory receptor OR2AR4. OR2AT4 is primarily found in the olfactory system, specifically in the olfactory epithelium, and is responsible for detecting and responding to specific odor molecules in the environment [8]. However, OR2AT4 expression is not restricted to olfactory tissues, as it can also be detected in other non-olfactory tissues, including the skin, hair follicles, and alveolar macrophages. [11,28]. In the skin, OR2AT4 is expressed in keratinocytes, and its ligand Sandalore promotes keratinocyte proliferation and migration, which are important processes in wound healing [9]. Recent studies have shown that OR2AT4 is activated by aromatic plant extracts and their constituents. *Cocos nucifera* flour extract stimulates keratinocyte migration in vitro, mediated by OR2AT4 [7]. Another study demonstrated that a lipid mixture composed of major lipid components extracted from *Chamaecyparis obtusa* upregulates antimicrobial peptides such as HBD-3 and LL-37 through OR2AT4, leading to accelerated skin wound healing in animal models [29]. We also established that the effects of PCE on cell proliferation and wound healing in HaCaT cells are mediated through the activation of OR2AT4, using Oxyphenylon, an OR2AT4 antagonist. The cotreatment of HaCaT cells with PCE and Oxyphenylon resulted in a significant reduction in cell proliferation and the promotion of wound healing. Furthermore, the number of HaCaT cells expressing Ki67 protein also significantly decreased, confirming that the action of PCE in keratinocytes is mediated through OR2AT4. Activation of OR2AT4 in keratinocytes triggers various intracellular signaling events, including the activation of pathways dependent on cAMP, increased calcium signaling, and phosphorylation of extracellular signal-regulated kinases (ERK1/2) and p38 mitogen-activated protein kinases (p38 MAPK) [30]. These molecular responses ultimately promote the proliferation and migration of human keratinocytes, thereby facilitating wound healing. In line with this, we confirmed that PCE stimulated the growth and movement of keratinocytes via OR2AT4 by inducing intracellular cAMP secretion, and observed that OR2AT4 inhibition by Oxyphenylon led to a decrease in PCE-induced cAMP secretion. These findings suggest that the activation of OR2AT4 by PCE promotes wound healing by triggering downstream signaling pathways, involving cAMP release.

Inflammation is a critical process that occurs in the early stages of wound healing. Upon injury, keratinocytes release cytokines such as IL-1, TNF-α, and IL-6, which initiate the inflammatory phase of wound healing. During the inflammation stage, macrophages not only phagocytose debris and bacteria but also release a range of cytokines, chemokines, and growth factors. Macrophages also facilitate the transition from inflammation to subsequent stages of wound healing, such as proliferation and remodeling, by secreting anti-inflammatory cytokines that help to resolve inflammatory responses [31]. However, if the inflammatory response becomes severe or is prolonged, it can lead to tissue damage, wound lesions, and serious diseases. To promote and improve wound healing, it is essential to control and reduce the inflammatory response, which involves the downregulation of pro-inflammatory gene expression [32]. Understanding the effect of PCE on inflammatory cytokines can provide insights into its potential role in modulating inflammatory responses during wound healing. Our study discovered that PCE demonstrated anti-inflammatory effects in THP-1 cells induced by LPS and HaCaT cells stimulated with TNF-α by suppressing the expression of genes related to inflammation. These findings are consistent with previous research, which demonstrates that the main components of PEO exhibit anti-inflammatory effects [16,17,33,34]. Patchouli alcohol has been shown to decrease the production of TNF-α, IL-6, IL-1β, prostaglandin E2, and nitric oxide in LPS-stimulated RAW264.7 cells by inhibiting ERK-mediated NF-κB activation [16,17]. Similarly, patchoulene epoxide and β-patchoulene exhibit potent anti-inflammatory activity by reducing inflammatory mediators and suppressing iNOS and COX-2 signaling pathways [33,34].

According to the findings of previous studies, it is highly likely that the anti-inflammatory signaling pathway of PCE involves inhibiting the NF-κB signaling pathway, which controls the expression of pro-inflammatory genes by reducing ERK1/2 phosphorylation and thus impeding ERK1/2 activation. Furthermore, a recent report indicated that activating OR2AT4 leads to a decrease in the expression of *CXCL8* and other pro-inflammatory cytokines that are dependent on NF-κB in alveolar macrophages, likely mediated through cAMP signaling [28]. cAMP can counteract the release of pro-inflammatory cytokines regulated by NF-κB [35]. Therefore, our findings suggest that the stimulation of OR2AT4 by PCE and the subsequent activation of cAMP signaling may play a role in its anti-inflammatory effects. Further research is required to clarify the molecular mechanisms by which PCE exerts its anti-inflammatory effects. Moreover, it is imperative to verify the wound-healing properties of PCE in vivo through human clinical trials to establish its potential as an effective promoter of wound healing.

## 5. Conclusions

We demonstrated that PCE derived from *P. cablin* leaves increases the proliferation of keratinocytes and accelerates wound healing. This is achieved through the activation of OR2AT4 and the subsequent increase in cAMP levels. Moreover, PCE exerts anti-inflammatory effects by reducing IL-6 and IL-8 expression in HaCaT keratinocytes and THP-1 differentiated macrophages. These findings indicate that PCE holds great potential as a therapeutic candidate for promoting wound healing.

## Data Availability

The datasets supporting the results of the current study are included in this article.
